# Temporal Trends in Incidence and Prevalence of Transthyretin Amyloid Cardiomyopathy in the United States

**DOI:** 10.1161/JAHA.125.047135

**Published:** 2026-06-23

**Authors:** Kevin M. Alexander, Noel R. Dasgupta, Jimmi Mathisen, Sidsel Gamborg Møller, Anders Tvistholm, Mathew S. Maurer

**Affiliations:** ^1^ Division of Cardiovascular Medicine, Stanford Amyloid Center Stanford University School of Medicine Stanford CA USA; ^2^ Indiana University School of Medicine Indianapolis IN USA; ^3^ Novo Nordisk A/S Søborg Denmark; ^4^ Cardiac Amyloidosis Program, Department of Medicine Columbia University Irving Medical Center, New York Presbyterian Hospital New York NY USA

**Keywords:** amyloidosis, cardiomyopathy, epidemiology, Cardiomyopathy

## Abstract

**Background:**

Advances in diagnosis and disease awareness have led to increased prevalence and incidence of transthyretin amyloid cardiomyopathy (ATTR‐CM), but current US population‐based epidemiologic data are lacking. This study aimed to estimate temporal trends and current prevalence and incidence of ATTR‐CM in the United States and to describe patient characteristics and the clinical burden.

**Methods:**

Data from the US Merative MarketScan Medicare Database (2010–2021) and the IQVIA PharMetrics Plus (2016–2023) claims database were assessed. An algorithm using codes for diagnoses and clinical drugs was used to identify patients with ATTR‐CM among individuals ≥18 years old. Incidence, prevalence, demographics, comorbidities, medication use, and clinical encounters were assessed annually in cross‐sectional cohorts.

**Results:**

The incidence and prevalence of ATTR‐CM became higher over time, particularly in men and older age groups. The incidence per 100 000 person‐years was 44.3 (95% CI, 39.4–49.2) in 2021 for the Medicare Database (individuals aged ≥65 years) and 5.4 (95% CI, 5.0–5.8) in 2022 for PharMetrics Plus (individuals aged ≥18 years); the prevalence per 100 000 individuals was 95.8 (95% CI, 89.2–102.4) in 2022 in the Medicare Database and 11.3 (95% CI, 10.8–11.8) in 2023 in PharMetrics Plus. The number of comorbidities and medication use in the year before diagnosis increased over time; the number of clinical encounters in the year before diagnosis was relatively stable.

**Conclusions:**

The incidence and prevalence of ATTR‐CM diagnoses have increased in the United States from 2010 to 2023, and the clinical burden among newly diagnosed individuals has become more complex.

## Graphical abstract

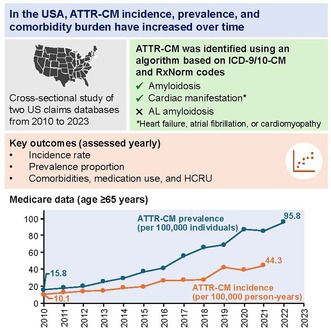

**Nonstandard Abbreviations and Acronyms**
AL amyloidosis: light chain amyloidosis
ATTR‐CM: transthyretin amyloid cardiomyopathy
TTR: transthyretin

**Clinical Perspective**

**What Is New?**

- Our study demonstrates that incidence and prevalence of transthyretin amyloid cardiomyopathy are increasing in the United States.
- A growing comorbidity burden and medication use in the year preceding the transthyretin amyloid cardiomyopathy diagnosis underscore the need for early diagnosis, because drug therapy is often more effective before significant end organ dysfunction occurs.

**What Are the Clinical Implications?**

- Future research should investigate strategies to improve diagnosis and treatment of transthyretin amyloid cardiomyopathy that consider age, sex, and race.

Transthyretin amyloid (ATTR) cardiomyopathy (ATTR‐CM) results from the deposition of TTR (transthyretin) amyloid fibrils in the myocardium. The disease can either be caused by wild‐type TTR protein sporadically misfolding, or by a genetic variation in the TTR gene (variant ATTR‐CM), which makes the protein prone to misfolding. More than 130 different TTR variants have been identified; the most common variants are Val50Met and Val142Ile, the latter being present in 3% to 4% of the Black population in the United States.^1^, ^2^, ^3^

ATTR‐CM is considered a rare disease, but the development of less invasive imaging techniques for the diagnosis of cardiomyopathies, coupled with increased disease awareness and the aging of the population, has led to a rise in the prevalence and incidence of diagnosed ATTR‐CM cases worldwide^4^, ^5^, ^6^, ^7^, ^8^; this suggests that the disease may be more accurately described as an underdiagnosed condition.

Globally, an estimated 20 per 100 000 adults have ATTR amyloidosis, of whom the majority have ATTR‐CM.^9^, ^10^, ^11^, ^12^ Furthermore, a 2008 autopsy study has found ATTR in 25% of heart specimens from adults older than 85 years of age.^13^ In the United States, the most recent population‐based estimate of the prevalence of ATTR‐CM is based on data collected during the period 2000 to 2012 from Medicare beneficiaries enrolled in the Medicare Fee‐for‐Service program who were aged 65 years or older.^5^ According to this study, the estimated prevalence of cardiac amyloidosis, which includes, but is not limited to, ATTR‐CM, is 55 per 100 000 individuals.^5^ It is noteworthy that this study included only individuals aged 65 years or older, yet ATTR‐CM can develop in individuals younger than 65 years of age.^5^ Thus, contemporary US epidemiologic population‐based data are currently lacking.

Given the paucity of current population‐based data on the epidemiology, patient characteristics, and clinical burden of ATTR‐CM in the United States, we set out to estimate the temporal trends and current prevalence and incidence of ATTR‐CM in 2 large US cohorts covering a broad age range. We further assessed over time the clinical burden of ATTR‐CM in terms of comorbidities, medication use, and health care resource use, and described the characteristics of patients with ATTR‐CM.

## METHODS

### Study Design and Data Source

This was a cross‐sectional study using data from 2 US claims databases, covering inpatient and outpatient data (see Figure S1). The Merative MarketScan Medicare Database includes data from individuals aged 65 years or older enrolled in Medicare Supplement and from 2020 also Medicare Advantage; data were from 2010 to 2021. The IQVIA PharMetrics Plus database includes fully adjudicated medical and pharmacy claims from commercially insured patients aged 18 years or older; data were from 2016 to 2023. In addition, data from the IQVIA Ambulatory Electronic Medical Records US database, linked to the IQVIA PharMetrics Plus database, were used to estimate the prevalence of ATTR‐CM stratified by race. Study periods and age ranges were based on data availability, and each database was assessed separately. Because this study was a retrospective analysis of secondary deidentified data not considered to be human subject research, it was exempt from institutional review board approval. The authors confirm that the data supporting the findings of this study are available within the article and its supplemental material. Because the data are commercially available and proprietary, the authors are unable to make the raw data available to other researchers.

### Study Populations

Individuals aged 18 years or older at index date and with at least 1 year of continuous enrollment in these health insurance providers before index were eligible for inclusion. Index date was defined as January 1 in every year in the study periods. Individuals with suspected light chain amyloidosis (AL amyloidosis) were excluded. Suspected AL amyloidosis was defined as having a diagnosis code for multiple myeloma, chronic myeloproliferative disease, or monoclonal gammopathy or having receipt of medication indicating treatment for AL amyloidosis (melphalan, bortezomib, thalidomide, daratumumab).

An algorithm using *International Classification of Diseases, Ninth/Tenth Revision, Clinical Modification* (*ICD‐9/10‐CM*) codes and RxNorm codes for clinical drugs was used to identify individuals with likely ATTR‐CM. ATTR‐CM was defined as having a diagnosis code both for amyloidosis and a cardiac manifestation (heart failure, atrial fibrillation, or cardiomyopathy), and no code indicating AL amyloidosis. A full list of codes used to define ATTR‐CM and AL amyloidosis can be found in Table S1. The frequency of specific diagnosis codes used to define ATTR‐CM, as well as the proportion of cases that were included based on atrial fibrillation and flutter as the only cardiac manifestation, are shown in Table S2.

### Outcomes

#### Incidence

Incident cases were identified among eligible individuals without ATTR‐CM and defined as individuals meeting the ATTR‐CM definition within a given year (Figure S1A). The incidence date was defined as the date when an incident case met the ATTR‐CM definition. Incidence rates were estimated as number of incident cases divided by total person‐years at risk for a given year; 95% CIs for the incidence rates were estimated using exact Poisson CIs. Incidence rates per 100 000 person‐years (95% CIs) were assessed annually in cross‐sectional cohorts, overall and stratified by age and sex. The denominators for the incidence rates, total person‐years at risk in a given year, were calculated by adding the days that all included individuals were at risk for developing ATTR‐CM, from index date (January 1) until the first occurrence of either ATTR‐CM diagnosis date, date of suspected AL amyloidosis, death, end of enrollment in health insurance, or 365 days from index. Incidence data were available up until and throughout 2021 (Medicare Database) or 2022 (PharMetrics Plus). To complement the yearly incidence rates and evaluate temporal trends, a Poisson regression was fitted with log link and log‐person‐years as an offset, modeling time as a continuous variable; the incidence rate ratio per 1‐year increase is reported.

#### Prevalence

Prevalent cases were identified as individuals meeting the ATTR‐CM definition before or on the index date each year (Figure S1B). Prevalence proportions per 100 000 individuals (95% CIs) were also assessed annually in cross‐sectional cohorts, overall and stratified by age, sex, and race. The denominator in the prevalence proportions was number of individuals in the database on a given index date fulfilling the eligibility criteria. Prevalence data were available up until 2022 (Medicare Database) or 2023 (PharMetrics Plus). The 95% CIs for prevalence were calculated using the exact binomial distribution. To complement the yearly prevalence proportions and to evaluate temporal trends, a binomial regression model with a logit link was fitted. Time was modeled as a continuous variable and prevalence odds ratio per 1‐year increase is reported.

#### Patient Characteristics and Health Care Resource Use

Patient characteristics and health care resource use were assessed annually among individuals with incident ATTR‐CM. Demographic characteristics were assessed on the incidence date. Medication use and clinical encounters (outpatient, inpatient, and emergency department visits) were assessed in the 365 days before the incidence date; comorbidities were assessed at any time before the incidence date. Data are presented as aggregates across calendar years (2010–2013, 2014–2017, and 2018–2021 for the Medicare Database, and 2016–2017, 2018–2020, and 2021–2022 for PharMetrics Plus).

### Statistical Analysis

Binary variables are summarized as counts and percentages. Count variables are grouped into categories and summarized as counts and percentages and also reported as median (25th–75th percentile [Q1–Q3]). Continuous variables are presented as mean±SD. Analyses were conducted in R, version 4.3.0.^14^

## RESULTS

### Study Population

The total number of individuals with incident ATTR‐CM across all years was 3801 in the Medicare Database and 3380 in PharMetrics Plus (Figure S2, Table 1). Age, sex, and geographical regions in the United States for the cohorts are shown in Table 1. The mean age of individuals with incident ATTR‐CM across all years was 79.7 years (SD 7.2) in the Medicare Database and 69.1 years (SD 12.5) in the PharMetrics Plus database (Table 1); the mean age appeared to increase throughout the years assessed in both databases (Table S3). In these cohorts, 57.2% and 60.0% of individuals were men, respectively. In the Medicare Database, data were primarily sourced from the East North Central region (29.3%) and the South Atlantic region (13.6%) of the United States (Table 1); the geographical distribution was more balanced in the PharMetrics Plus database. The characteristics of the cohort identified from PharMetrics Plus linked with Ambulatory Electronic Medical Records are shown in Table S4.

**Table 1:** Demographics and Geography of Incident Cases and Corresponding At‐Risk Population (Medicare Database and PharMetrics Plus)

	Medicare Database (2010–2021)			PharMetrics Plus (2016–2023)		
	Cases (all years)	Cases (2021)	At risk (2021)	Cases (all years)	Cases (2022)	At risk (2022)
Number of individuals, N	3801	314	734 819	3380	766	15 936 707
Demographics						
Age, y	79.7±7.2	80.0±6.7	76.7±7.2	69.1±12.5	70.2±13.0	46.4±16.1
Men	2173 (57.2)	187 (59.6)	335 636 (45.7)	2028 (60.0)	465 (60.7)	7 812 901 (49.0)
US region						
East North Central region	1113 (29.3)	190 (60.5)	458 422 (62.4)	865 (25.6)	213 (27.8)	2 943 251 (18.5)
East South Central region	86 (2.3)	8 (2.5)	31 311 (4.3)	222 (6.6)	56 (7.3)	1 338 098 (8.4)
Mid‐Atlantic region	435 (11.4)	16 (5.1)	38 138 (5.2)	404 (12.0)	89 (11.6)	1 759 378 (11.0)
Mountain region	161 (4.2)	13 (4.1)	39 309 (5.3)	91 (2.7)	22 (2.9)	694 000 (4.4)
New England region	257 (6.8)	6 (1.9)	6025 (0.8)	310 (9.2)	55 (7.2)	964 846 (6.1)
Pacific region	338 (8.9)	3 (1.0)	15 143 (2.1)	529 (15.7)	107 (14.0)	1 334 314 (8.4)
South Atlantic region	516 (13.6)	53 (16.9)	99 654 (13.6)	517 (15.3)	126 (16.4)	3 005 094 (18.9)
West North Central region	188 (4.9)	17 (5.4)	24 629 (3.4)	226 (6.7)	52 (6.8)	1 571 566 (9.9)
West South Central region	247 (6.5)	8 (2.5)	21 803 (3.0)	210 (6.2)	46 (6.0)	2 304 217 (14.5)
Other	460 (12.1)	0 (0.0)	385 (0.1)	6 (0.2)	0 (0.0)	21 943 (0.1)

Values are mean±SD or n (%).

### Incidence and Prevalence of ATTR‐CM

**Figure 1:**
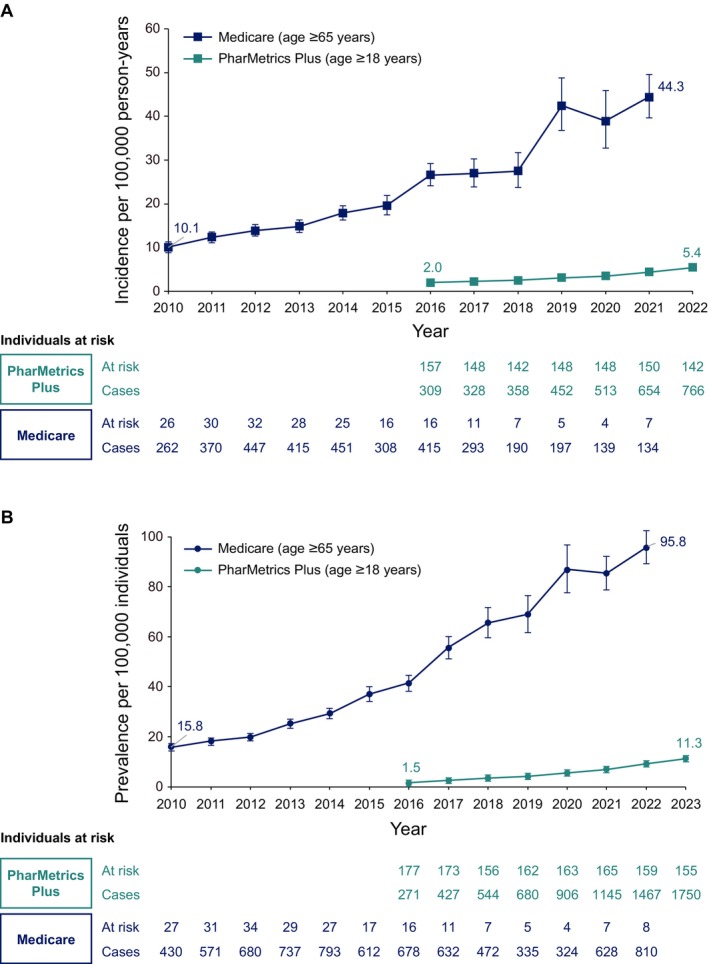
Annual incidence (A) and prevalence (B) of ATTR‐CM 2010 to 2023 (Medicare Database and PharMetrics Plus). At‐risk count is reported per 100 000 individuals. Incidence is reported as rates (number of cases per person‐year). Prevalence is reported as proportions (number of cases per number of individuals in the total population). Error bars represent 95% CIs. CIs are not visible at the shown scale for PharMetrics Plus data points. Underlying data are shown in Table S4. ATTR‐CM indicates transthyretin amyloid cardiomyopathy.

Across both databases, the annual incidence and prevalence of ATTR‐CM became higher over time (Figure 1; underlying data are shown in Table S5). The incidence of ATTR‐CM per 100 000 person‐years was 10.1 (95% CI, 8.9–11.4) in 2010 and 44.3 (95% CI, 39.6–49.5) in 2021 for the Medicare Database (individuals ≥65 years of age); for PharMetrics Plus (individuals aged ≥18 years) it was 2.0 (95% CI, 1.8–2.2) in 2016 and 5.4 (95% CI, 5.0–5.8) in 2022. A similar trend was observed for prevalence: the prevalence per 100 000 individuals was 15.8 (95% CI, 14.4–17.4) in 2010 and 95.8 (95% CI, 89.3–102.6) in 2022 in the Medicare Database; in the PharMetrics Plus database it was 1.5 (95% CI, 1.4–1.7) in 2016 and 11.3 (95% CI, 10.8–11.9) in 2023. The incidence rate ratio for a 1‐year increase was 1.15 (95% CI, 1.13–1.16) in the Medicare Database and 1.19 (95% CI, 1.17–1.21) in the PharMetrics Plus database. The prevalence odds ratio for a 1‐year increase in the Medicare Database was 1.17 (95% CI, 1.17–1.18) and in the PharMetrics Plus database it was 1.30 (95% CI, 1.28–1.31).

**Figure 2:**
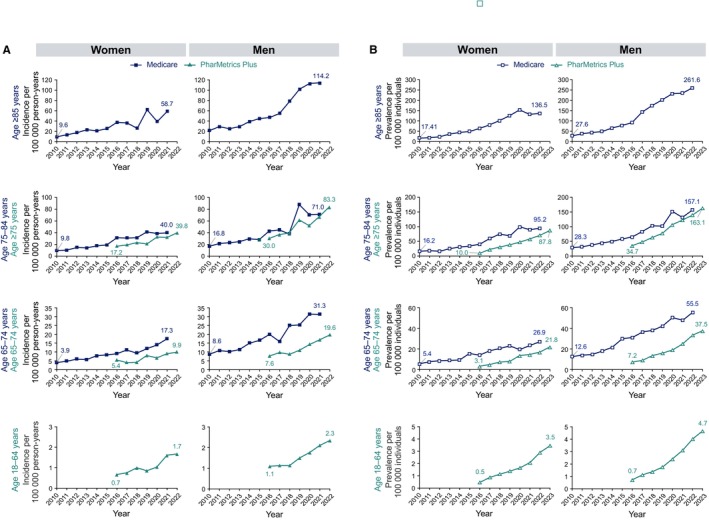
Annual incidence (A) and prevalence (B) by sex and age (Medicare Database and PharMetrics Plus). Data for individuals aged ≥85 years and 18–64 years are available only from the Medicare Database and PharMetrics Plus, respectively. For those aged ≥75 years, 2 separate groups are required to accommodate the available data in the Medicare Database and in PharMetrics Plus. Incidence is reported as rate and prevalence is measured as proportions (number of cases per number of individuals in the total population).

Increases in incidence and prevalence were observed across all the sex and age subgroups studied, but in general incidence and prevalence were higher and increased more in men than in women and in older than in younger age groups (Figure 2).

Data to compare the prevalence by race were available only from 2019 to 2023. The prevalence among Black individuals could not be estimated before 2019 because of the low number of these individuals with ATTR‐CM in the databases. Similarly, incidence by race could not be determined. Based on the limited available data, the prevalence of ATTR‐CM increased over time at a higher rate in Black individuals than in White individuals (Figure S3).

### Comorbidities

**Figure 3:**
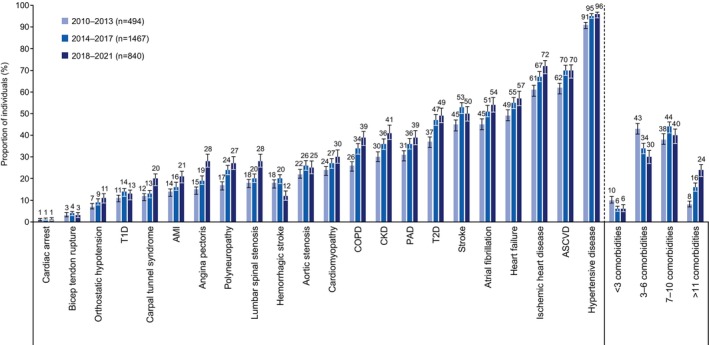
Prevalence (crude proportions and 95% CIs) of comorbidities observed before index date,* by year groups (Medicare Database). *The diagnosis codes given on index day were not included. AMI indicates acute myocardial infarction; ASCVD, atherosclerotic cardiovascular disease; CKD, chronic kidney disease; COPD, chronic obstructive pulmonary disease; PAD, peripheral artery disease; T1D, type 1 diabetes; T2D, type 2 diabetes.

Between 2010 and 2021, there was a trend toward larger proportions of individuals experiencing comorbidities over time in the Medicare Database (Figure 3). Hypertensive disease, atherosclerotic cardiovascular disease (stroke, acute myocardial infarction, or peripheral artery disease), and ischemic heart disease were the 3 most commonly recorded comorbidities between 2010 and 2021, with hypertensive disease consistently affecting the greatest number of individuals (90.8%–95.6%). The incidence of comorbidities generally associated with ATTR‐CM, such as lumbar spinal stenosis, polyneuropathy, and carpal tunnel syndrome, increased over the study period; changes from the first to the last study period were 17.8% to 28.3% for lumbar spinal stenosis, 16.7% to 27.3% for polyneuropathy, and 11.5% to 19.5% for carpal tunnel syndrome.

The proportion of individuals with more than 11 comorbidities increased from 8.3% (95% CI, 6.9%–9.7%) in the 2010 to 2013 group to 23.8% (95% CI, 20.9%–26.7%) in the 2018 to 2021 group; at the same time, the proportion of individuals with 3–6 comorbidities decreased from 43.1% (95% CI, 40.6%–45.6%) to 30.1% (95% CI, 27.0%–33.2%) (Figure 3). To assess if this observation is confounded by age and sex, direct standardization by age and sex was performed, using the same age categories as before (65–74 years, 75–84 years, ≥85 years) and the entire cohort with incident ATTR‐CM as standard population. Adjusting for age and sex did not affect the proportion of individuals with <3, 3 to 6, 7 to 10, and ≥11 comorbidities (Table S6), supporting the hypothesis that the number of comorbidities is increasing over time, regardless of differences in age and sex distributions.

All trends were generally similar in the PharMetrics Plus database (Table S7).

### Medication Use and Clinical Encounters

**Figure 4:**
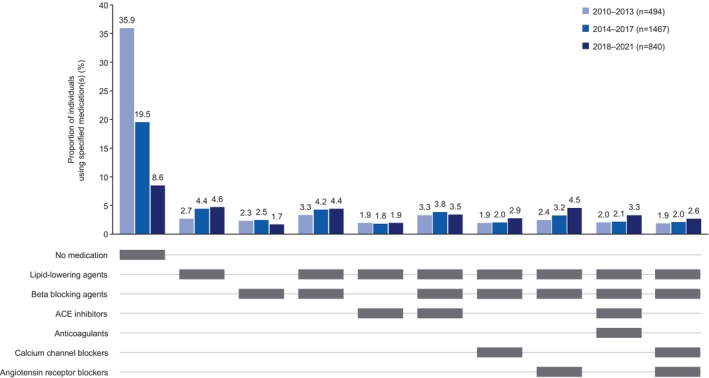
Most frequent drug combinations in the year before index date in the 2010 to 2013 cohort* (Medicare Database). *Drug use was measured as the patient having observed ≥1 prescription for any of the medications in the year before index date. The top 10 most frequent drug combinations for the cohort 2010–2013 were taken as a reference group, and the proportion of individuals with the same drug combinations for the 2014–2017 and 2018–2021 cohorts have been plotted. ACE indicates angiotensin‐converting enzyme.

In the Medicare Database, lipid‐lowering and beta blocking agents were the most used medications in the 365 days before individuals were classified with incident ATTR‐CM (Table 2). Over time, medication use in the 365 days before classification as an individual with incident ATTR‐CM became higher in the Medicare Database for all medication types, except for angiotensin‐converting enzyme inhibitors and diuretics, which were stable (Table 2); similarly, the proportion of individuals in receipt of common drug combinations generally increased with the year groups in the Medicare Database (Figure 4). At the same time, the proportions of individuals receiving no medication became lower over time, from 35.9% (95% CI, 33.4%–38.3%) in 2010 to 2013 to 8.6% (95% CI, 6.7%–10.5%) in 2018 to 2021 (Figure 4); age‐ and sex‐adjusted proportions for the groups without medication prescriptions in the index year are shown in Table S8. There was a trend toward increased use of drug combination regimens across year groups; adjusting for age and sex did not change the results.

**Table 2:** Medication Use in the Year Before Index* Date Across Cohorts by Year Group (Medicare Database)

Medication	Year group		
	2010–2013 (n=1494)	2014–2017 (n=1467)	2018–2021 (n=840)
Beta blocking agents	621 (42)	758 (52)	512 (61)
Angiotensin‐converting enzyme inhibitors	389 (26)	423 (29)	225 (27)
Angiotensin receptor blockers	248 (17)	334 (23)	266 (32)
Anticoagulants	285 (19)	371 (25)	308 (37)
Calcium channel blockers	359 (24)	445 (30)	290 (35)
Diuretics	240 (16)	265 (18)	144 (17)
Lipid‐lowering agents	625 (42)	814 (55)	573 (68)

Values are n (%).

* At least 1 prescription recorded in the year before index.

In the 365 days before being classified as an individual with incident ATTR‐CM, a considerable proportion of individuals in the Medicare Database had outpatient, inpatient, and emergency department visits, with >90% of individuals having had ≥6 outpatient visits and ≥60% having had between 1 and 5 emergency department visits (Table S9). Across year groups, the proportion of individuals with outpatient and emergency department visits remained relatively stable, whereas the proportion of individuals with inpatient visits appeared to become smaller over time. Among individuals with ≥1 clinical encounters, the median (Q1–Q3) number of any kind of visit was 30 (17–49), 31 (18–50), and 32 (19–49) for the 2010 to 2013, 2014 to 2017, and 2018 to 2021 cohorts, respectively. Clinical encounters for the PharMetrics Plus cohort are shown in Table S10.

## DISCUSSION

This study provides contemporary estimates of the incidence and prevalence of ATTR‐CM in the United States, demonstrating a marked increase in both metrics over the study time frame. In individuals aged 65 years or older identified from the Medicare Database, the incidence of ATTR‐CM was >4‐fold higher in 2021 than in 2010, and the prevalence was 6‐fold higher in 2022 than in 2010. Trends of increasing incidence and prevalence were also observed for individuals aged 18 years or older from the PharMetrics Plus cohort. This rise over time is likely due to improved diagnosis and increased disease awareness, possibly driven by the availability of a disease‐specific treatment and more widespread use of less invasive diagnostic techniques.^5^, ^15^

For individuals aged 65 years or older identified from the Medicare Database, the incidence and prevalence of ATTR‐CM was markedly higher than in US epidemiology data previously reported by Gilstrap et al. in patients with cardiac amyloidosis (including, but not limited to, ATTR‐CM) within the same age bracket (incidence: 44.3 per 100 000 person‐years in 2021 versus 17 per 100 000 person‐years in 2012; prevalence: 95.8 per 100 000 individuals in 2022 versus 55 per 100 000 individuals in 2012), corroborating an increasing incidence and prevalence over time.^5^ Epidemiologic assessments from other parts of the world highlight that the estimated prevalence of ATTR‐CM varies between countries and age groups.^7^, ^8^, ^16^, ^17^ Globally, it is estimated that ∼20 per 100 000 adults have ATTR amyloidosis.^9^, ^10^, ^11^, ^12^

Notably, both incidence and prevalence of ATTR‐CM were greater in men than in women and in older than in younger individuals, suggesting that male sex and older age are risk factors for the disease.^18^ Underdiagnosis in women could be due to sex bias, differences in symptom presentation, and a historical lack of research focused on women in cardiovascular diseases, but further research is needed to clarify this.^19^ Although the absolute numbers remain lower among younger people (<65 years old), the rising trend in incidence and prevalence in this group is noteworthy. Furthermore, we found that ATTR‐CM was considerably more prevalent in Black individuals than in White individuals. However, the linkage of PharMetrics Plus and Ambulatory Electronic Medical Records data required for this analysis resulted in a relatively small pool of individuals in which to identify an already rare disease. In our study, Black and White individuals had similar mean ages (mean age range across 2016–2023: 45.8–49.9 years for Black individuals versus 47.5–49.3 years for White individuals) and similar proportions of men (range across 2016–2023: 40.1%–41.3% versus 45.5%–46.1%, respectively), indicating that the higher prevalence among Black individuals compared with White individuals is not driven by age and sex differences in the at‐risk populations. This underscores a need for strategies to improve diagnosis and treatment of ATTR‐CM that consider age, sex, and race, for example improvements in care options or clinical trials that represent these at‐risk subgroups.

A substantial proportion of individuals had comorbidities, and the number of comorbidities recorded before diagnosis of ATTR‐CM increased over the study duration. Given that hypertension, atherosclerotic cardiovascular disease, and ischemic heart disease were particularly prevalent among individuals with likely ATTR‐CM in our cohorts, patients with these comorbidities with other red flags should be considered for ATTR‐CM screening in clinical practice. A rise in the overall clinical burden of ATTR‐CM is also supported by an increase in the use of medications, often as combination regimens, suggesting that individuals may manage complex treatment regimens, possibly in relation to signs and symptoms caused by ATTR‐CM before the diagnosis, although this cannot be established from the available data. This growing clinical burden of disease likely causes additional costs for health care systems, requiring more resources and specialized care.

### Strengths and Limitations

These analyses used data from large databases, each geographically covering the whole of the United States, over a long time period and across all adult age ranges. This allowed for comprehensive estimates of the current epidemiology of ATTR‐CM, as well as an assessment of trends since 2010. Furthermore, extensive data on health care resource use, in addition to medication usage and detailed information on comorbidities, enabled the characterization of individuals in the year before their ATTR‐CM diagnosis. Our study also had limitations. The algorithm for identifying ATTR‐CM cases is based on diagnosis codes (but not specific codes for ATTR‐CM) and medication use; although it is not formally validated, it allowed for identification of patients with likely ATTR‐CM, with some uncertainty arising from methodological constraints. The algorithm can identify only people who are likely to have ATTR‐CM; therefore, individuals identified cannot be considered as patients with confirmed ATTR‐CM. This broad definition may have led to the inclusion of some false positives, possibly overestimating the number of ATTR‐CM cases. Conversely, ATTR‐CM remains an underdiagnosed disease, and it is possible that true positives were incorrectly excluded. Although the proportion of men in this study (∼60%) is lower than that usually seen in clinical practice (∼80%), the majority of ATTR‐CM cases were nonetheless observed in men. It is noteworthy that ascertainment programs have found similar numbers of men and women with the disease,^20^ which differs from what is seen in referral centers and aligns with what has been observed in this study. Furthermore, the algorithm for identifying ATTR‐CM cases does not distinguish between hereditary and wild‐type ATTR‐CM. In addition, patients with ATTR‐CM may have monoclonal gammopathy as a comorbidity, but these patients would have been excluded using the algorithm, meaning a number of ATTR‐CM cases could have been overlooked. Another limitation is inherent to the databases used: PharMetrics Plus covers commercially and self‐insured individuals, as well as a small proportion of Medicare enrollees, whereas the Medicare Database captures a subset of Medicare‐enrolled individuals who were registered with Medicare Supplement (all years) or Medicare Advantage (from 2020). Furthermore, there was a high turnover of the at‐risk populations during the study periods. Consequently, our results may not be fully representative of the general population in the United States. Lastly, it is also important to note that this study was not designed to investigate specific causes of the increases in incidence and prevalence of the disease.

## CONCLUSIONS

In this US database study, the incidence and prevalence of ATTR‐CM became higher over the study period across all age ranges, with higher rates in men than in women and among older than younger individuals. The burden before diagnosis has also grown more complex, primarily driven by the need to manage a wide variety of comorbidities and medications. These trends highlight a need for timely diagnosis and comprehensive disease management strategies, as highlighted in the 2023 Expert Consensus Decision Pathway on Comprehensive Multidisciplinary Care for the Patient With Cardiac Amyloidosis published by the American College of Cardiology.^21^ Development of further noninvasive diagnostic tools to aid the early diagnosis as well as additional disease‐specific treatments are essential to improve outcomes for patients with ATTR‐CM.

## Sources of Funding

This study was funded by Novo Nordisk A/S, Søborg, Denmark.

## Disclosures

Kevin M. Alexander has received consulting income from Alexion/AstraZeneca, Alnylam, Bayer, BridgeBio, Novo Nordisk, and Pfizer. Noel R. Dasgupta has received consulting income from Akcea, Alnylam, Eidos, Intellia, Novo Nordisk, and Pfizer and her institution received clinical trial funding from Alexion, Alnylam, Eidos, Intellia, and Ionis/AstraZeneca. Jimmi Mathisen, Sidsel Gamborg Møller, and Anders Tvistholm are employees of Novo Nordisk A/S. Mathew S. Maurer reports grant support from the National Institutes of Health (R01HL139671 and R01AG081582). He has received consulting income from Alnylam, AstraZeneca, Eidos, Intellia, Ionis, Novo Nordisk, and Pfizer, and his institution has received clinical trial funding from Alnylam, Attralus, Eidos, Ionis, Pfizer, and Prothena.

## Supporting information

Tables S1–S10
Figures S1–S3
