# Supplementary material for: Temporal Trends in Incidence and Prevalence of Transthyretin Amyloid Cardiomyopathy in the United States
Source: J Am Heart Assoc. 2026 Jun 23;15(13):e047135. doi: 10.1161/JAHA.125.047135 (PMC13477393; doi:10.1161/JAHA.125.047135)
Supplement: Supplementary file 1 [file JAH3-15-e047135-s001.pdf]

Supplemental Material

Table S1. ICD-9/10-CM Codes and Medications Used to Define ATTR-CM and AL Amyloidosis

ICD-10-CM	Diagnosis	ICD-9-CM	Diagnosis
**Amyloidosis (inclusion criteria)**			
E85	Amyloidosis	277.3*	Amyloidosis
E85.0	Non-neuropathic heredofamilial amyloidosis		
E85.1	Neuropathic heredofamilial amyloidosis		
E85.2	Heredofamilial amyloidosis, unspecified		
E85.4	Organ-limited amyloidosis		
E85.8	Other amyloidosis		
E85.82	Wild-type transthyretin-related (ATTR) amyloidosis		
E85.89	Other amyloidosis		
E85.9	Amyloidosis unspecified		
**Cardiac manifestation (inclusion criteria)**			
Heart failure			
I50*	Heart failure	428*	Heart failure
Cardiomyopathy			
I42	Cardiomyopathy	425	Cardiomyopathy
I42.0	Dilated cardiomyopathy	425.1	Hypertrophic cardiomyopathy
I42.1	Obstructive hypertrophic cardiomyopathy	425.2	Obscure cardiomyopathy of Africa
I42.2	Other hypertrophic cardiomyopathy	425.4	Other primary cardiomyopathies
I42.5	Other restrictive cardiomyopathy	425.7	Nutritional and metabolic cardiomyopathy
I42.8	Other cardiomyopathies	425.8	Cardiomyopathy in other diseases classified elsewhere
I42.9	Cardiomyopathy, unspecified		
I43*	Cardiomyopathy in diseases classified elsewhere		
Atrial fibrillation or flutter			
I48*	Atrial fibrillation and flutter	427.3*	Atrial fibrillation and flutter
**AL amyloidosis (exclusion criteria)**			
C90.0*	Multiple myeloma	203.0*	Multiple myeloma
C90.1*	Plasma cell leukemia	203.1*	Plasma cell leukemia
D47.1	Chronic myeloproliferative disease	238.76	Myelofibrosis with myeloid metaplasia

D47.2	Monoclonal gammopathy (of undetermined significance [MGUS])	273.1	Monoclonal paraproteinemia
E85.81	Light chain (AL) amyloidosis		
**Medications**			
Bortezomib			
Daratumumab			
Melphalan			
Thalidomide			

AL amyloidosis indicates light chain amyloidosis; ATTR, transthyretin amyloid; ATTR-CM, transthyretin amyloid cardiomyopathy; ICD-9/10-CM, International Classification of Diseases, Ninth/Tenth Revision, Clinical Modification.

*Includes the code itself and all descendant codes.

Table S2. Frequency of Specific Diagnosis Codes for Definition of ATTR-CM Cohorts, and Proportion of Cases That Were Included Based on Atrial Fibrillation And Flutter as the Only Cardiac Manifestation at Index (Medicare Data)

Disease type	Subtype	Code	Name	Women (n= 1628)	Men (n= 2173)	Total (n= 3801)
Amyloidosis	NA	277.3	Amyloidosis	16 (1.0%)	15 (0.7%)	31 (0.8%)
		277.30	Amyloidosis, unspecified	371 (22.8%)	557 (25.6%)	928 (24.4%)
		277.31	Familial Mediterranean fever	74 (4.5%)	43 (2.0%)	117 (3.1%)
		277.39	Other amyloidosis	573 (35.2%)	777 (35.8%)	1350 (35.5%)
		E85.0	Non-neuropathic heredofamilial amyloidosis	24 (1.5%)	24 (1.1%)	48 (1.3%)
		E85.1	Neuropathic heredofamilial amyloidosis	22 (1.4%)	12 (0.6%)	34 (0.9%)
		E85.2	Heredofamilial amyloidosis, unspecified	1 (0.1%)	9 (0.4%)	10 (0.3%)
		E85.4	Organ-limited amyloidosis	392 (24.1%)	535 (24.6%)	927 (24.4%)
		E85.8	Other amyloidosis	33 (2.0%)	46 (2.1%)	79 (2.1%)
		E85.82	Wild-type transthyretin-related (ATTR) amyloidosis	5 (0.3%)	21 (1.0%)	26 (0.7%)
		E85.89	Other amyloidosis	21 (1.3%)	24 (1.1%)	45 (1.2%)
		E85.9	Amyloidosis, unspecified	192 (11.8%)	246 (11.3%)	438 (11.5%)
Cardiomyopathy	Atrial fibrillation	427.31	Atrial fibrillation	685 (42.1%)	1079 (49.7%)	1764 (46.4%)
		427.32	Atrial flutter	117 (7.2%)	249 (11.5%)	366 (9.6%)
		I48.0	Paroxysmal atrial fibrillation	209 (12.8%)	320 (14.7%)	529 (13.9%)
		I48.1	Persistent atrial fibrillation	71 (4.4%)	118 (5.4%)	189 (5.0%)
		I48.11	Longstanding persistent atrial fibrillation	6 (0.4%)	14 (0.6%)	20 (0.5%)
		I48.19	Other persistent atrial fibrillation	16 (1.0%)	34 (1.6%)	50 (1.3%)
		I48.2	Chronic atrial fibrillation	104 (6.4%)	204 (9.4%)	308 (8.1%)
		I48.20	Chronic atrial fibrillation, unspecified	26 (1.6%)	58 (2.7%)	84 (2.2%)

		I48.21	Permanent atrial fibrillation	18 (1.1%)	29 (1.3%)	47 (1.2%)
		I48.3	Typical atrial flutter	13 (0.8%)	39 (1.8%)	52 (1.4%)
		I48.4	Atypical atrial flutter	13 (0.8%)	33 (1.5%)	46 (1.2%)
		I48.91	Unspecified atrial fibrillation	301 (18.5%)	489 (22.5%)	790 (20.8%)
		I48.92	Unspecified atrial flutter	56 (3.4%)	134 (6.2%)	190 (5.0%)
	Cardiomyopathy	425.1	Hypertrophic cardiomyopathy	15 (0.9%)	29 (1.3%)	44 (1.2%)
		425.2	Obscure cardiomyopathy of Africa	2 (0.1%)	4 (0.2%)	6 (0.2%)
		425.4	Other primary cardiomyopathies	261 (16.0%)	527 (24.3%)	788 (20.7%)
		425.7	Nutritional and metabolic cardiomyopathy	57 (3.5%)	107 (4.9%)	164 (4.3%)
		425.8	Cardiomyopathy in other diseases classified elsewhere	25 (1.5%)	49 (2.3%)	74 (1.9%)
		I42.0	Dilated cardiomyopathy	36 (2.2%)	84 (3.9%)	120 (3.2%)
		I42.1	Obstructive hypertrophic cardiomyopathy	13 (0.8%)	12 (0.6%)	25 (0.7%)
		I42.2	Other hypertrophic cardiomyopathy	16 (1.0%)	37 (1.7%)	53 (1.4%)
		I42.5	Other restrictive cardiomyopathy	10 (0.6%)	43 (2.0%)	53 (1.4%)
		I42.8	Other cardiomyopathies	41 (2.5%)	107 (4.9%)	148 (3.9%)
		I42.9	Cardiomyopathy, unspecified	92 (5.7%)	248 (11.4%)	340 (8.9%)
		I43	Cardiomyopathy in diseases classified elsewhere	66 (4.1%)	190 (8.7%)	256 (6.7%)
	Heart failure	428	Congestive heart failure, unspecified	675 (41.5%)	967 (44.5%)	1642 (43.2%)
		428.1	Left heart failure	83 (5.1%)	149 (6.9%)	232 (6.1%)
		428.2	Systolic heart failure, unspecified	52 (3.2%)	85 (3.9%)	137 (3.6%)
		428.21	Acute systolic heart failure	67 (4.1%)	94 (4.3%)	161 (4.2%)
		428.22	Chronic systolic heart failure	81 (5.0%)	163 (7.5%)	244 (6.4%)

		428.23	Acute on chronic systolic heart failure	61 (3.7%)	143 (6.6%)	204 (5.4%)
		428.3	Diastolic heart failure, unspecified	119 (7.3%)	135 (6.2%)	254 (6.7%)
		428.31	Acute diastolic heart failure	88 (5.4%)	102 (4.7%)	190 (5.0%)
		428.32	Chronic diastolic heart failure	123 (7.6%)	170 (7.8%)	293 (7.7%)
		428.33	Acute on chronic diastolic heart failure	120 (7.4%)	133 (6.1%)	253 (6.7%)
		428.4	Combined systolic and diastolic heart failure, unspecified	16 (1.0%)	32 (1.5%)	48 (1.3%)
		428.41	Acute combined systolic and diastolic heart failure	13 (0.8%)	30 (1.4%)	43 (1.1%)
		428.42	Chronic combined systolic and diastolic heart failure	33 (2.0%)	55 (2.5%)	88 (2.3%)
		428.43	Acute on chronic combined systolic and diastolic heart failure	34 (2.1%)	77 (3.5%)	111 (2.9%)
		428.9	Heart failure, unspecified	127 (7.8%)	185 (8.5%)	312 (8.2%)
		I50.1	Left ventricular failure, unspecified	23 (1.4%)	43 (2.0%)	66 (1.7%)
		I50.20	Unspecified systolic (congestive) heart failure	42 (2.6%)	96 (4.4%)	138 (3.6%)
		I50.21	Acute systolic (congestive) heart failure	38 (2.3%)	82 (3.8%)	120 (3.2%)
		I50.22	Chronic systolic (congestive) heart failure	62 (3.8%)	164 (7.5%)	226 (5.9%)
		I50.23	Acute on chronic systolic (congestive) heart failure	49 (3.0%)	118 (5.4%)	167 (4.4%)
		I50.30	Unspecified diastolic (congestive) heart failure	86 (5.3%)	121 (5.6%)	207 (5.4%)

		I50.31	Acute diastolic (congestive) heart failure	54 (3.3%)	78 (3.6%)	132 (3.5%)
		I50.32	Chronic diastolic (congestive) heart failure	125 (7.7%)	182 (8.4%)	307 (8.1%)
		I50.33	Acute on chronic diastolic (congestive) heart failure	95 (5.8%)	135 (6.2%)	230 (6.1%)
		I50.40	Unspecified combined systolic (congestive) and diastolic (congestive) heart failure	13 (0.8%)	32 (1.5%)	45 (1.2%)
		I50.41	Acute combined systolic (congestive) and diastolic (congestive) heart failure	10 (0.6%)	35 (1.6%)	45 (1.2%)
		I50.42	Chronic combined systolic (congestive) and diastolic (congestive) heart failure	22 (1.4%)	70 (3.2%)	92 (2.4%)
		I50.43	Acute on chronic combined systolic (congestive) and diastolic (congestive) heart failure	41 (2.5%)	94 (4.3%)	135 (3.6%)
		I50.810	Right heart failure, unspecified	6 (0.4%)	8 (0.4%)	14 (0.4%)
		I50.811	Acute right heart failure	2 (0.1%)	1 (0.0%)	3 (0.1%)
		I50.812	Chronic right heart failure	2 (0.1%)	5 (0.2%)	7 (0.2%)
		I50.813	Acute on chronic right heart failure	3 (0.2%)	3 (0.1%)	6 (0.2%)
		I50.814	Right heart failure due to left heart failure	1 (0.1%)	NA	1 (0.0%)
		I50.82	Biventricular heart failure	5 (0.3%)	4 (0.2%)	9 (0.2%)
		I50.84	End stage heart failure	1 (0.1%)	1 (0.0%)	2 (0.1%)
		I50.89	Other heart failure	2 (0.1%)	6 (0.3%)	8 (0.2%)
		I50.9	Heart failure, unspecified	295 (18.1%)	430 (19.8%)	725 (19.1%)

Atrial fibrillation only	NA	NA	NA	442 (27.1%)	506 (23.3%)	948 (24.9%)

ATTR indicates transthyretin amyloid; ATTR-CM, transthyretin amyloid cardiomyopathy; NA, not applicable.

Table S3. Mean age (SD) in years at the beginning of each year for incident cases

Year	Medicare	PharMetrics Plus
**2010**	79.0 (7.1)	NA
**2011**	79.6 (7.5)	NA
**2012**	79.4 (7.1)	NA
**2013**	79.7 (7.2)	NA
**2014**	79.1 (7.3)	NA
**2015**	79.9 (7.3)	NA
**2016**	80.0 (7.1)	67.0 (11.8)
**2017**	80.0 (6.9)	68.4 (11.3)
**2018**	79.5 (7.1)	68.6 (12.2)
**2019**	80.6 (7.0)	69.5 (12.0)
**2020**	79.9 (7.4)	69.5 (12.4)
**2021**	80.0 (6.7)	68.8 (13.1)
**2022**	NA	70.2 (13.0)
**2023**	NA	NA

NA indicates not available; SD, standard deviation.

Table S4. Demographics and Geographical Distribution of Incident Cases and Corresponding At-Risk Population Across All Databases

	Medicare Database			PharMetrics Plus			PharMetrics Plus/Ambulatory EMR		
	**Cases (all years)**	**Cases (2021)**	**At risk (2021)**	**Cases (all years)**	**Cases (2022)**	**At risk (2022)**	**Cases (all years)**	**Cases (2022)**	**At risk (2022)**
Number of individuals, N	3801	314	734,819	3380	766	15,936,707	598	133	1,921,782
Demographics									
Age, years	79.7 ± 7.2	80.0 ± 6.7	76.7 ± 7.2	69.1 ± 12.5	70.2 ± 13.0	46.4 ± 16.1	68.9 ± 11.7	69.2 ± 12.0	48.9 ± 16.1
Men	2173 (57.2)	187 (59.6)	335,636 (45.7)	2028 (60.0)	465 (60.7)	7,812,901 (49.0)	361 (60.4)	87 (65.4)	874,244 (45.5)
US region									
East North Central region	1113 (29.3)	190 (60.5)	458,422 (62.4)	865 (25.6)	213 (27.8)	2,943,251 (18.5)	117 (19.6)	29 (21.8)	335,414 (17.5)
East South Central region	86 (2.3)	8 (2.5)	31,311 (4.3)	222 (6.6)	56 (7.3)	1,338,098 (8.4)	58 (9.7)	14 (10.5)	244,962 (12.7)
Mid-Atlantic region	435 (11.4)	16 (5.1)	38,138 (5.2)	404 (12.0)	89 (11.6)	1,759,378 (11.0)	81 (13.5)	18 (13.5)	278,635 (14.5)
Mountain region	161 (4.2)	13 (4.1)	39,309 (5.3)	91 (2.7)	22 (2.9)	694,000 (4.4)	14 (2.3)	3 (2.3)	45,887 (2.4)
New England region	257 (6.8)	6 (1.9)	6025 (0.8)	310 (9.2)	55 (7.2)	964,846 (6.1)	65 (10.9)	15 (11.3)	123,963 (6.5)
Pacific region	338 (8.9)	3 (1.0)	15,143 (2.1)	529 (15.7)	107 (14.0)	1,334,314 (8.4)	73 (12.2)	12 (9.0)	104,884 (5.5)
South Atlantic region	516 (13.6)	53 (16.9)	99,654 (13.6)	517 (15.3)	126 (16.4)	3,005,094 (18.9)	113 (18.9)	27 (20.3)	398,506 (20.7)
West North Central region	188 (4.9)	17 (5.4)	24,629 (3.4)	226 (6.7)	52 (6.8)	1,571,566 (9.9)	39 (6.5)	8 (6.0)	214,330 (11.2)
West South Central region	247 (6.5)	8 (2.5)	21,803 (3.0)	210 (6.2)	46 (6.0)	2,304,217 (14.5)	37 (6.2)	7 (5.3)	171,289 (8.9)
Other	460 (12.1)	0 (0.0)	385 (0.1)	6 (0.2)	0 (0.0)	21,943 (0.1)	1 (0.2)	0 (0.0)	3912 (0.2)

EMR indicates electronic medical record. Values are mean ± SD or n (%).

Table S5. Annual Incidence and Prevalence ATTR-CM From 2010 to 2023 (Medicare Database and PharMetrics Plus)

Database	Year	Incidence per 100,000 person-years (95% CI)	Prevalence per 100,000 individuals (95% CI)
**Medicare Database**	2010	10.1 (8.9–11.4)	15.8 (14.4–17.4)
	2011	12.3 (11.1–13.6)	18.2 (16.7–19.7)
	2012	13.8 (12.6–15.2)	19.9 (18.4–21.4)
	2013	14.8 (13.4–16.3)	25.2 (23.4–27.1)
	2014	17.9 (16.3–19.6)	29.4 (27.3–31.5)
	2015	19.6 (17.4–21.9)	37.1 (34.2–40.1)
	2016	26.6 (24.1–29.2)	41.5 (38.4–44.7)
	2017	26.9 (23.9–30.2)	55.6 (51.4–60.2)
	2018	27.5 (23.7–31.7)	65.6 (59.8–71.8)
	2019	42.4 (36.7–48.7)	68.9 (61.7–76.6)
	2020	38.9 (32.7–45.9)	86.9 (77.7–96.9)
	2021	44.3 (39.6–49.5)	85.4 (78.8–92.3)
	2022	–	95.8 (89.3–102.6)
**PharMetrics Plus**	2016	2.0 (1.8–2.2)	1.5 (1.4–1.7)
	2017	2.2 (2.0–2.5)	2.5 (2.2–2.7)
	2018	2.5 (2.3–2.8)	3.5 (3.2–3.8)
	2019	3.1 (2.8–3.4)	4.2 (3.9–4.5)
	2020	3.5 (3.2–3.8)	5.6 (5.2–5.9)
	2021	4.4 (4.0–4.7)	7.0 (6.6–7.4)
	2022	5.4 (5.0–5.8)	9.2 (8.7–9.7)
	2023	–	11.3 (10.8–11.9)

ATTR-CM indicates transthyretin amyloid cardiomyopathy.
Incidence is reported as rates (number of cases per person-years). Prevalence is reported as proportions (number of cases per number of individuals in the total population).

Table S6. Proportion (95% CI) of Individuals With Comorbidities (<3, 3–6, 7–10, ≥11), Adjusted for Age and Sex with Entire ATTR-CM Incident Population as Standard Population (Medicare Database)

**Year group, % (95% CI)**	**Number of comorbidities**			
	**<3**	**3–6**	**7–10**	**≥11**
**2010–2013**	10.2 (8.7–11.8)	43.1 (40.6–45.6)	38.4 (36.0–40.9)	8.3 (6.9–9.7)
**2014–2017**	6.1 (4.9–7.4)	33.8 (31.4–36.2)	43.8 (41.3–46.4)	16.2 (14.3–18.1)
**2018–2021**	6.6 (4.9–8.3)	30.7 (27.6–33.8)	39.7 (36.4–43.0)	22.9 (20.2–25.7)

ATTR-CM indicates transthyretin amyloid cardiomyopathy.

Table S7. Prevalence of Comorbidities Observed in the Year Before Index Date, by Year Groups (PharMetrics Plus)

	**Year group**		
	**2016–2017 (n=637)**	**2018–2019 (n=1323)**	**2021–2022 (n=1420)**
Acute myocardial infarction	67 (11)	211 (16)	244 (17)
Angina pectoris	73 (11)	262 (20)	294 (21)
Aortic stenosis	55 (9)	142 (11)	149 (10)
ASCVD	230 (36)	575 (43)	623 (44)
Atrial fibrillation	254 (40)	578 (44)	647 (46)
Bicep tendon rupture	6 (1)	9 (1)	18 (1)
Cardiac arrest	5 (1)	18 (1)	16 (1)
Cardiomyopathy	157 (25)	398 (30)	456 (32)
Carpal tunnel syndrome	36 (6)	137 (10)	219 (15)
Chronic kidney disease	200 (31)	514 (39)	562 (40)
COPD	127 (20)	327 (25)	324 (23)
Heart failure	283 (44)	709 (54)	776 (55)
Hemorrhagic stroke	58 (9)	111 (8)	85 (6)
Hypertensive disease	528 (83)	1171 (89)	1263 (89)
Ischemic heart disease	299 (47)	732 (55)	786 (55)
Lumbar spinal stenosis	42 (7)	182 (14)	230 (16)
Orthostatic hypotension	48 (8)	100 (8)	112 (8)
Peripheral artery disease	89 (14)	241 (18)	260 (18)
Polyneuropathy	87 (14)	226 (17)	281 (20)
Stroke	135 (21)	311 (24)	349 (25)
T1D	31 (5)	89 (7)	74 (5)
T2D	206 (32)	501 (38)	552 (39)
**Any comorbidity***			
<3 comorbidities	155 (24)	200 (15)	228 (16)
3–6 comorbidities	323 (51)	620 (47)	616 (43)
7–10 comorbidities	141 (22)	414 (31)	450 (32)
≥11 comorbidities	18 (3)	89 (7)	126 (9)

ASCVD indicates atherosclerotic cardiovascular disease; ATTR-CM, transthyretin amyloid cardiomyopathy; COPD, chronic obstructive pulmonary disease; CV, cardiovascular; T1D, type 1 diabetes; T2D, type 2 diabetes.
Values are n (%).
*The diagnosis codes given on index day were not included.

Table S8. Proportion (95% CI) of Individuals in Each Year Group With No Medication Prescriptions in the Year Before Index Date (Medicare Database)

Year group	Crude proportion (95% CI)	Age- and sex-adjusted proportion (95% CI)
**2010–2013**	35.9 (33.4–38.3)	35.9 (33.5–38.4)
**2014–2017**	19.5 (17.5–21.5)	19.5 (17.5–21.5)
**2018–2021**	8.6 (6.7–10.5)	8.7 (6.8–10.6)

ATTR-CM indicates transthyretin amyloid cardiomyopathy.
Adjustments for age and sex were done by direct standardization using all incident ATTR-CM cases as the standard population and the following age categories: 65–74 years, 75–84 years, ≥85 years.

Table S9. Clinical Encounters in the Year Before Index Date for Individuals with ATTR-CM (Medicare Database)

	Year group		
	**2010–2013 (n=1494)***	**2014–2017 (n=1467)***	**2018–2021 (n=840)***
**Emergency room visit**			
0	514 (34)	464 (32)	315 (38)
1 to 5	901 (60)	915 (62)	475 (57)
≥6	79 (5)	88 (6)	50 (6)
Median (Q1–Q3)*	2 (1–3)	2 (1–4)	2 (1–4)
**Inpatient visit**			
0	526 (35)	563 (38)	385 (46)
1 to 5	661 (44)	656 (45)	356 (42)
≥ 6	307 (21)	248 (17)	99 (12)
Median (Q1–Q3)*	3 (1–8)	2 (1–6)	2 (1–5)
**Outpatient visit**			
0	13 (1)	20 (1)	3 (0)
1 to 5	121 (8)	113 (8)	52 (6)
≥6	1360 (91)	1334 (91)	785 (93)
Median (Q1–Q3)*	22 (12–35)	22 (13–35)	23 (14–37)
**Other visit**†			
0	650 (44)	576 (39)	314 (37)
1 to 5	453 (30)	490 (33)	277 (33)
≥6	391 (26)	401 (27)	249 (30)
Median (Q1–Q3)*	5 (2–12)	4 (2–11)	5 (2–14)
**Any visit**‡			
0	3 (0)	6 (0)	1 (0)
1 to 5	61 (4)	51 (3)	21 (3)
≥6	1430 (96)	1410 (96)	818 (97)
Median (Q1–Q3)*	30 (17–49)	31 (18–50)	32 (19–49)

ATTR-CM indicates transthyretin amyloid cardiomyopathy; Q, quartile.
Values are n (%) or median (Q1–Q3).
*Q1–Q3 calculated among those with ≥1 encounter. †"Other visit" includes home visits, nonhospital institution visits, telehealth visits, and ambulance visits. Because "Other visit" is an aggregation of multiple different visits, multiple visits could occur on the same day if the underlying visits occurred on the same day, for instance if a person had both a telehealth visit and an ambulance visit on the same day. ‡Because "Any visit" is an aggregation of all other visit types, multiple visits could occur on the same day if the underlying visits occurred on the same day.

Table S10. Clinical Encounters in the Year Before Index Date for Individuals with ATTR-CM (PharMetrics)

	Year group		
	**2016–2017 (n=637)***	**2018–2020 (n=1323)***	**2021–2022 (n=1420)***
**Emergency room visit**			
0	253 (40)	522 (39)	593 (42)
1 to 5	330 (52)	719 (54)	766 (54)
≥6	54 (8)	82 (6)	61 (4)
Median (Q1–Q3)*	2 (1–4)	2 (1–3)	2 (1–3)
**Inpatient visit**			
0	277 (43)	637 (48)	738 (52)
1 to 5	277 (43)	544 (41)	555 (39)
6+	83 (13)	142 (11)	127 (9)
Median (Q1–Q3)*	2 (1–5)	2 (1–4)	2 (1–4)
**Outpatient visit**			
0	4 (1)	12 (1)	17 (1)
1 to 5	42 (7)	84 (6)	91 (6)
≥6	591 (93)	1227 (93)	1312 (92)
Median (Q1–Q3)*	25 (14–43)	24 (14–41)	24 (13–39)
**Other visit**[†]			
0	456 (72)	919 (69)	957 (67)
1 to 5	133 (21)	343 (26)	409 (29)
≥6	48 (8)	61 (5)	54 (4)
Median (Q1–Q3)*	2 (1–6)	1 (1–4)	1 (1–3)
**Any visit**[‡]			
0	2 (<1)	8 (1)	10 (1)
1 to 5	32 (5)	57 (4)	67 (5)
≥6	603 (95)	1258 (95)	1343 (95)
Median (Q1–Q3)*	29 (17–52)	28 (17–46)	27 (15–45)

ATTR-CM indicates transthyretin amyloid cardiomyopathy; Q, quartile.
Values are n (%) or median (Q1–Q3).
*Q1–Q3 calculated among those with ≥1 encounter. [†]"Other visit" includes home visits, nonhospital institution visits, telehealth visits, and ambulance visits. Because "Other visit" is an aggregation of multiple different visits, multiple visits could occur on the same day if the underlying visits occurred on the same day, for instance if a person had both a telehealth visit and an ambulance visit on the same day. [‡]Because "Any visit" is an aggregation of all other visit types, multiple visits could occur on the same day if the underlying visits occurred on the same day.

Figure S1. Study Design to Determine A) Annual Incidence and B) Annual (Day 1) Point Prevalence

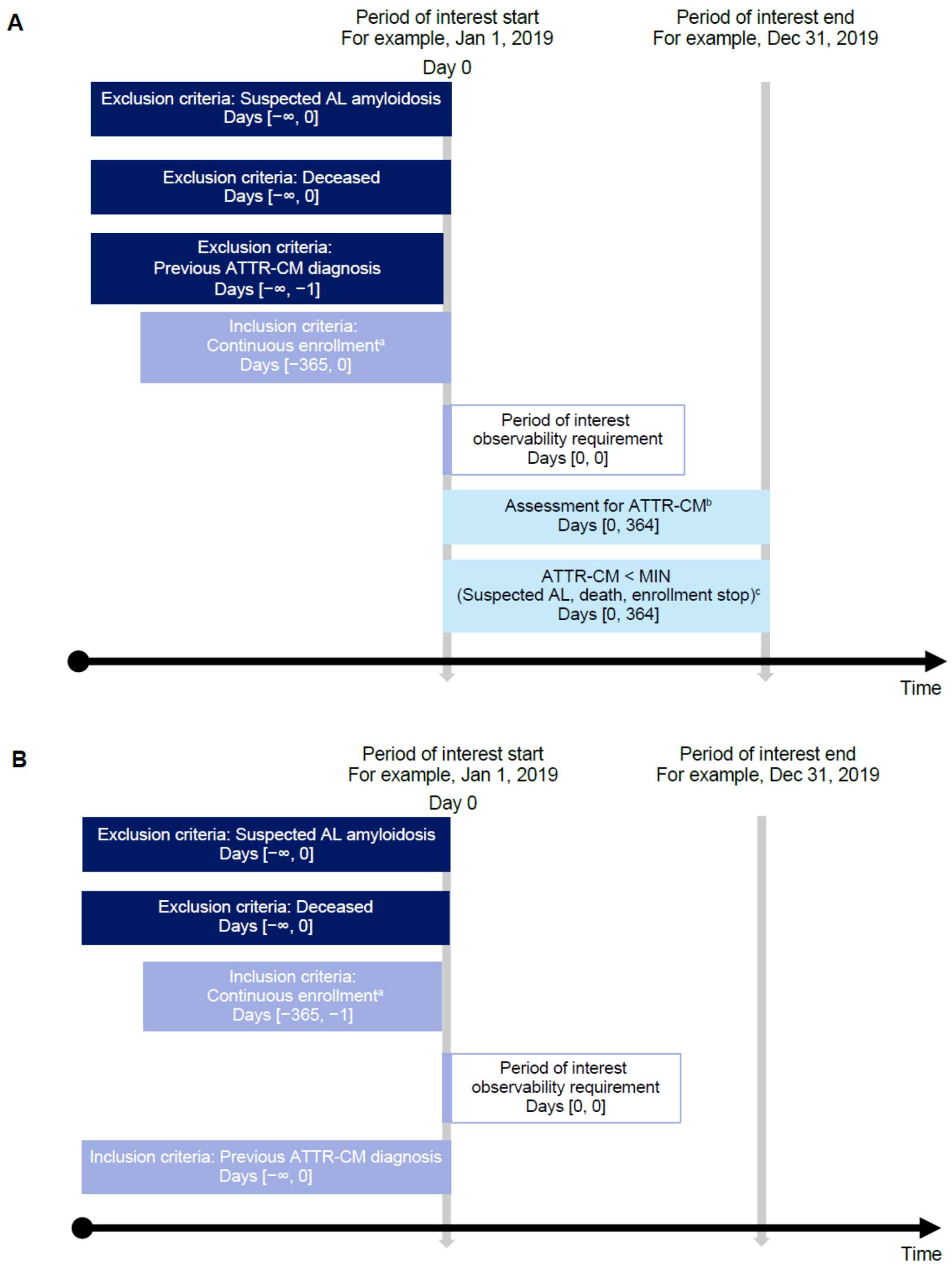

[a]Assessed using different values: 180, 365, 730, 1460, 2920; [b]First day at which case criteria is fulfilled. It is not necessary to have the amyloidosis code and the cardiac implication code within the period of interest, ie, one code can be registered before the start of the period of interest. [c]ATTR-CM case criteria need to be fulfilled prior to suspected AL amyloidosis diagnosis, death, or stop of enrollment.

AL indicates amyloid light chain; ATTR-CM, transthyretin amyloid cardiomyopathy.

Figure S2. Patient Attrition Medicare Database

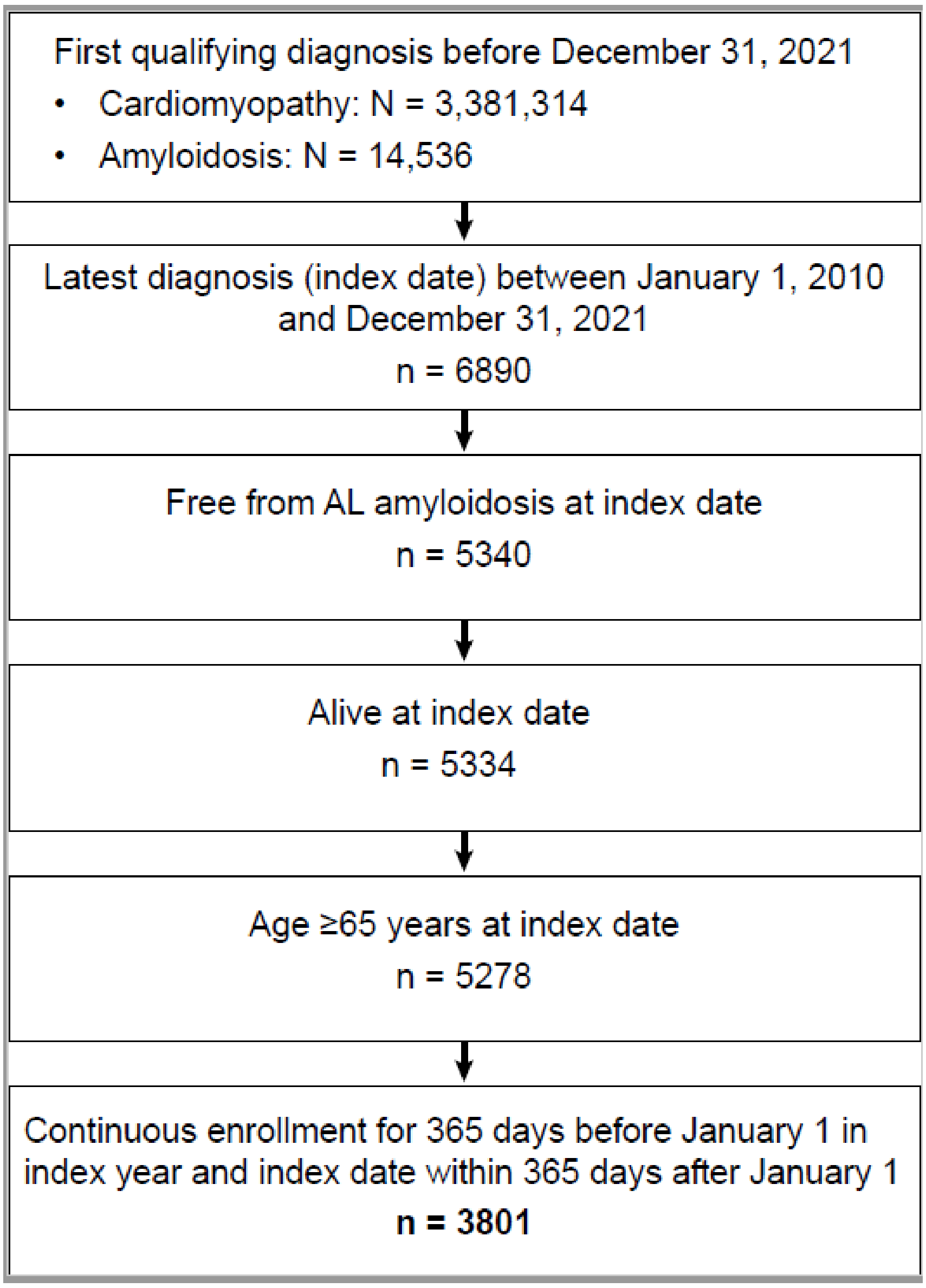

Figure S3. Prevalence of ATTR-CM by Race

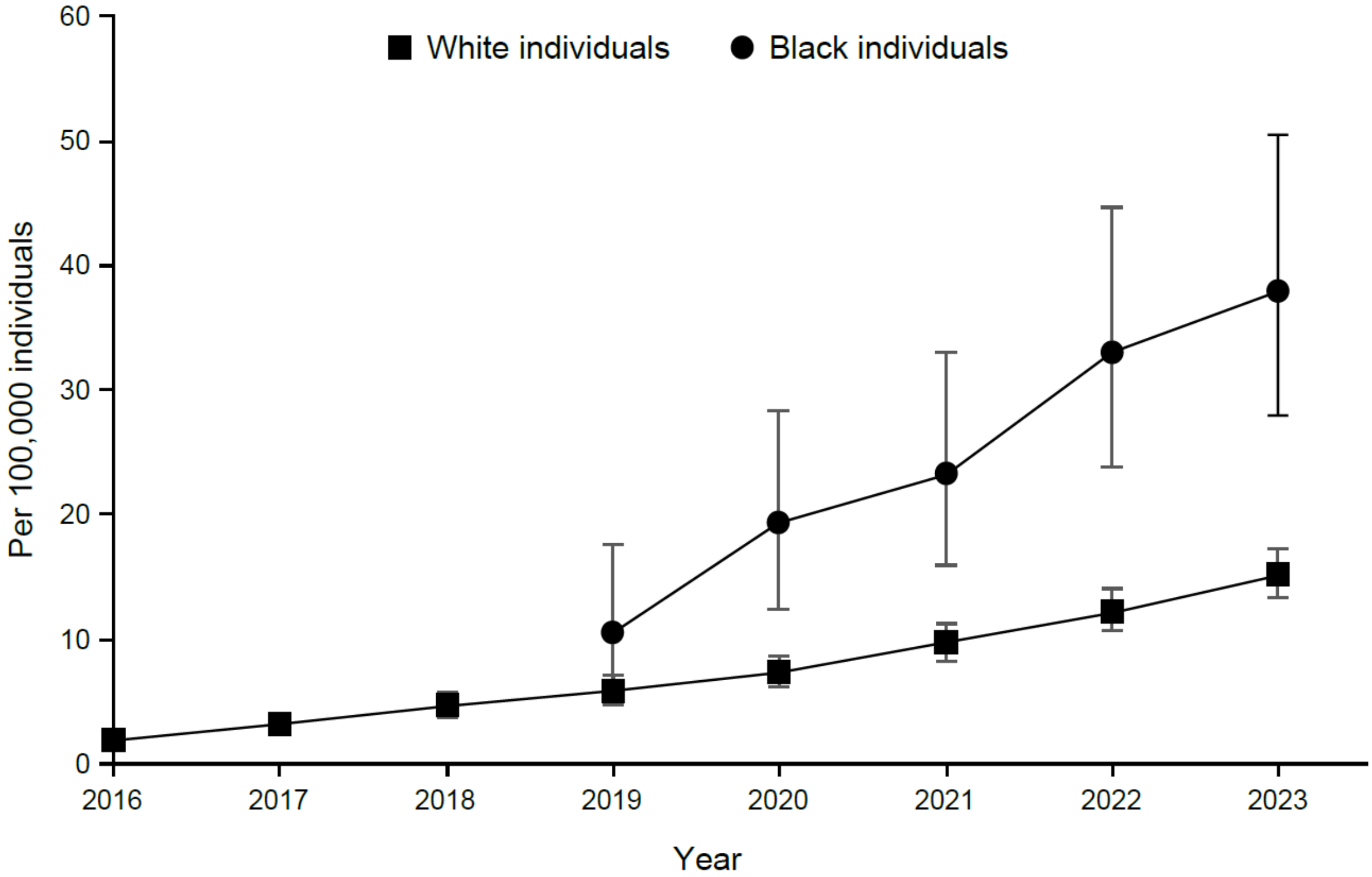

Data were obtained from the PharMetrics Plus/Ambulatory EMR database. Prevalence is measured as a Day 1 prevalence proportion where Day 1 refers to January 1 in the year of interest. Error bars represent 95% CIs. Data for years with fewer than 10 cases are not shown. ATTR-CM indicates transthyretin amyloid cardiomyopathy; EMR, electronic medical record.
